# Severe food insecurity among middle-aged and older adults in India: Insights from the Longitudinal Aging Study in India

**DOI:** 10.1016/j.gfs.2024.100822

**Published:** 2025-03

**Authors:** Ravi Sadhu, Rockli Kim, S.V. Subramanian

**Affiliations:** a Department of Global Health and Population, Harvard T.H. Chan School of Public Health, Boston, MA, USA; b Division of Health Policy and Management, College of Health Science, Korea University, Seoul, South Korea; c Interdisciplinary Program in Precision Public Health, Department of Public Health Sciences, Graduate School of Korea University, Seoul, South Korea; d Harvard Center for Population and Development Studies, Cambridge, MA, USA; e Department of Social and Behavioral Sciences, Harvard T. H. Chan School of Public Health, Boston, MA, USA

**Keywords:** Severe food insecurity, Social determinants, Geography, Adults, India

## Abstract

Severe food insecurity (SFI) is a critical concern in India, yet few studies have highlighted national and state trends among Indian adults. Utilizing data from 63,525 adults aged 45 and above from the Longitudinal Aging Study in India (2017-18), we present noteworthy geographic and sociodemographic patterns in the distribution of SFI. We estimate the national prevalence to be 6.2% (95% CI: 5.6, 6.8). Madhya Pradesh had the highest prevalence of SFI (10.3%), followed by Jharkhand, Tamil Nadu, and Bihar. Conversely, Nagaland, Goa, Lakshadweep, and Arunachal Pradesh had the lowest prevalence (all under 2.0%). Living in a rural area, being widowed, belonging to a Scheduled Caste, and being Muslim were positively associated with reporting SFI. Additionally, those in the middle and richer household monthly per capita expenditure quintiles, with 6–9 years of schooling, who resided in the country's northern and northeastern regions, or who had never worked for more than three months in their lifetime, were less likely to report SFI. We also find that geography and socioeconomic status synergistically affect SFI. Specifically, adults with lower levels of education were at a greater risk of SFI in rural areas, relative to urban areas, and the Central and East regions of India, relative to the North. Our findings highlight the need for robust state-level policies to ensure equitable access to affordable, high-quality food for all Indian adults, particularly for those belonging to at-risk groups.

## Highlights

• We investigate severe food insecurity in India, which is underexplored.
• About 6% of Indian adults aged 45 and above were found to be severely food insecure.
• Severe food insecurity was the highest in states in central and eastern India.
• Caste, religion, socioeconomic and marital status are pertinent correlates.

## 1 Introduction

Though India is one of the world's leading food producers today (Pingali et al., 2017), it is ranked 105th out of 127 countries for hunger (Global Hunger Index, 2024). Some answers to this paradox lie in long-standing challenges with inefficient food supply chains, interstate and intrastate inequities in food access, and the pervasive adverse impact of climate change on crop and livestock yields (Balaji and Arshinder, 2016; Pingali et al., 2019; Praveen and Sharma, 2020; Ray et al., 2019). Recently, the COVID-19 pandemic and its aftermath have likely significantly exacerbated food insecurity due to rising food prices, inadequate government provision of food relief, and compromised livelihoods for workers in the informal sector who form the bulk of India's workforce (Emediegwu and Nnadozie, 2023; Jaacks et al., 2021; Sinha, 2021).

Due to these long-standing and contemporary challenges, now is a pivotal time to survey the landscape of food insecurity in India in its extreme form: severe food insecurity (SFI). Global evidence corroborates that SFI damages older adults' physical and mental health, quality of life, and well-being (Selvamani et al., 2023; Smith et al., 2021, 2023). In concordance with global trends, two nationally representative studies in India indicate that severely food-insecure adults aged 60 and above are at a greater risk of malnutrition and lower cognitive function (Kandapan et al., 2023; Roy, 2023). Since SFI is an influential upstream determinant of physical and mental health among older adults in India, further attention is warranted to identify which adult groups are at risk and where.

Despite the need, only a few studies have highlighted trends in SFI (broadly defined) across the life course and among diverse socioeconomic groups in India. Subramanian et al. examined a metric of extreme food insecurity among children and infants called “Zero-food” (Subramanian et al., 2023). They found that although the prevalence of “Zero-food” marginally decreased from 1993, it remained high at 17.8% in 2021, with significant state-level variation. Kandapan et al. estimate the prevalence of SFI among older Indian adults to be 5.0% (Kandapan et al., 2023). Mandal and Pradhan stratified adults aged 45 and older by migration status and found that SFI among migrants was 6.2% and non-migrants was 6.6% (Mandal and Pradhan, 2024). In addition to these nationally representative studies, cohort studies from different regions of India with varying sample sizes have estimated an SFI prevalence below 25% (Ayiraveetil et al., 2020; Ganpule et al., 2023; Langer et al., 2024). These studies have highlighted some at-risk groups: women, people with physical illnesses, and individuals from low-income backgrounds.

Our study addresses four underexplored gaps in the aforementioned literature. First, previous national findings on SFI among adults are primarily restricted to older adults. We provide national and state-representative data using a more extensive age demographic of middle-aged and older adults aged 45 and above. Second, we describe adult SFI trends by state/union territory (UT), revealing significant geographic trends in SFI. As is the case of food deprivation among infants and children (Subramanian et al., 2023), SFI among adults in India is likely to be strongly patterned along regional and state contours. This is likely due to area-specific historical, economic, and human development trends in different regions and states of India (Kurian, 2000). Furthermore, the operationalization and budgeting of employment and welfare schemes are decentralized and differ by state capacity.

Third, we present the social and demographic groups that may be especially vulnerable to SFI. Nationally representative studies on food insecurity among Indian adults have pointed to pertinent correlates-monthly expenditure, rural residence, education, caste, and geographic region (Fong, 2023; Kandapan et al., 2023). We aim to explore whether similar graded social patterns exist for a more extreme measure of food insecurity. Finally, socioeconomic and spatial factors likely influence SFI in India. By examining whether the associations between education level, wealth quintile, and SFI change by urban-rural residence and geographic region, we highlight a new intersectional understanding of socioeconomic status, regionality, and SFI in India.

## 2 Methodology

### 2.1 Sampling design and ethical considerations

Wave 1 Data were collected from April 2017 to December 2018, excluding the state of Sikkim, for which data collection was conducted from 2020 to 2021. Wave 1 of the Longitudinal Aging Study in India (LASI) covered a panel sample of 73,396 individuals aged 45 and above and their spouses from all states and union territories of India (International Institute for Population Sciences, 2020). LASI involved a multistage stratified area probability cluster sampling method that is representative at the national level. In rural and urban areas, households were chosen in three and four stages, respectively (Perianayagam et al., 2022). The first stage for rural and urban regions consisted of selecting primary sampling units in each state/UT: sub-districts (*tehsils/talukas*). The second stage involved selecting villages in rural areas and wards in urban areas in the chosen subdistricts. In rural areas, households were then selected from villages. In urban areas, households were selected from urban census blocks.

The individual response rate from the age-eligible households selected was 87% (Bloom et al., 2021). Ethical approval was obtained from various ethical boards, including the Indian Council of Medical Research. Written informed consent was collected from respondents (Perianayagam et al., 2022). Since our study involved the analysis of de-identified secondary data, we did not seek ethical approval for this study.

### 2.2 Severe food insecurity

We constructed our measure of SFI using two of the five items of LASI's food security module (Table 1). The two items ask the following: whether respondents in the last 12 months 1) were hungry but did not eat because there was not enough food in their household, and 2) did not eat for a whole day because there was not enough food in their household (International Institute for Population Sciences, 2020). Responses to these two questions were aggregated into a single measure; those who answered “yes” to one or both questions were classified as severely food insecure.

**Table 1:** Survey items of the Food Insecurity Experience Scale (left) and the food security module of the Longitudinal Aging Study of India (right).

FIES (Food Insecurity Experience Scale)	LASI Wave 1 (2017-18)
During the last 12 months, was there a time when, because of lack of money or other resources: 1. You were worried you would not have enough food to eat? 2. You were unable to eat healthy and nutritious food? 3. You ate only a few kinds of foods? 4. You had to skip a meal? 5. You ate less than you thought you should? 6. Your household ran out of food? 7. You were hungry but did not eat? 8. You went without eating for a whole day?	FO230. In the last 12 months, did you ever reduce the size of your meals or skip meals because there was not enough food at your household? 1. Yes 2. No FO231. In the last 12 months, did you eat enough food of your choice? Please exclude fasting/food related restrictions due to religious or health related reason. 1. Yes 2. No FO232. In the last 12 months, were you hungry but didn't eat because there was not enough food at your household? Please exclude fasting/food related restrictions due to religious or health related reasons. 1. Yes 2. No FO233. In the past 12 months did you ever not eat for a whole day because there was not enough food at your household? Please exclude fasting/food related restrictions due to religious or health related reasons. 1. Yes 2. No FO234. Do you think that you have lost weight in the last 12 months because there was not enough food at your household? 1. Yes 2. No

These two items have been adapted from Items 7 and 8 of the Food Insecurity Experience Scale (FIES) (Table 1). The FIES is an international eight-item reference scale created by the Food and Agriculture Organization of the United Nations (Cafiero et al., 2018). It has been tested in 153 countries and territories, making it a valid tool for cross-country and cross-region comparisons of mild and severe individual and household food insecurity (Cafiero et al., 2018). In the FIES, respondents are considered to live in severely food insecure households if they give an affirmative response to either or both items: During the last 12 months, was there a time when, because of lack of money or other resources: a) You were hungry but did not eat?; and 2) You went without eating for a whole day? Our definition and measurement of SFI follow the standard methodology of the FIES.

The FIES and adapted versions have been used in several studies in India (Chyne et al., 2017; Ganpule et al., 2023; Maxfield, 2020; Onori et al., 2021; Reshmi et al., 2019). Overall, studies using the FIES and modified versions in India have demonstrated cross-cultural compatibility across diverse locations, ethnic, caste, social, and age groups (McKay et al., 2023). Considering that these two items have been psychometrically tested and validated as a measure of SFI in the Indian context (Sethi et al., 2017), we determined that they should form our composite measure. This two-item measure has also been used in recent LASI studies and justified for similar reasons (Kandapan et al., 2023; Roy, 2023). As supporting evidence, the two items demonstrated acceptable internal consistency (Cronbach's alpha = 0.79).

### 2.3 Correlates

For this study, we considered pertinent demographic variables, including age group (45–54 years/55–64 years/65–74 years/75+ years), gender (Men/Women), marital status (Married/Widowed/Other), religion (Hindu/Muslim/Christian/Other), caste (Upper Castes/Scheduled Castes/Scheduled Tribes/Other Backward Classes), geographic region (North/Northeast/East/Central/West/South), residence (Rural/Urban), and migration status (Lived in the current area of residence for their whole life/Lived in the current area for ten years or more/Lived in the current location for less than ten years). In addition, we included education (No education/1–5 years of schooling/6–9 years of schooling/Ten years or more), household monthly per capita expenditure (Poorest/Poor/Middle/Richer/Richest), and working status (Never worked for more than three months/Currently not working/Presently working), as socioeconomic correlates of interest.

### 2.4 Participants and inclusion criteria

**Fig. 1:**
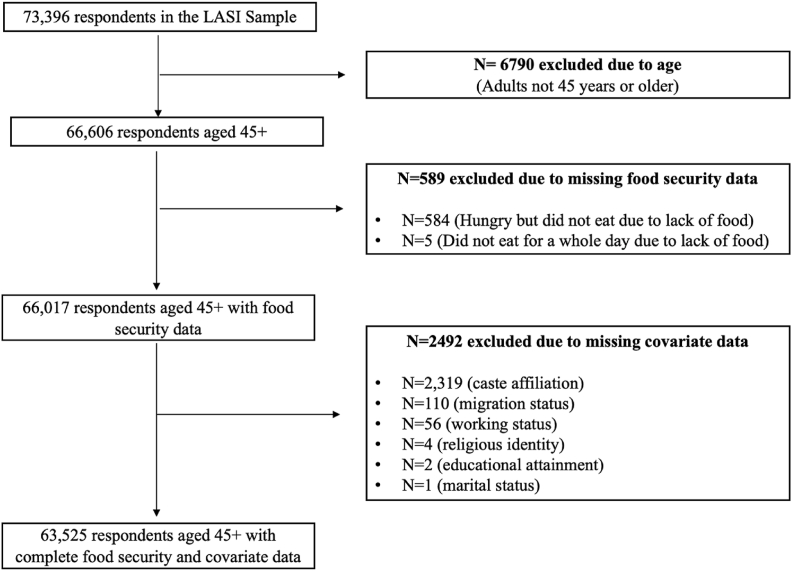
Flow chart showing the exclusion criteria and final sample size of the study population, LASI (2017-18).

Individuals below the age of 45 (n = 6790) and those who had not answered both questions on food insecurity (n = 589) were dropped (Fig. 1). The percentage of missing data on food insecurity across all social and demographic covariates was less than 1%, barring caste. Additionally, respondents with missing covariate data were also dropped (n = 2492). Upon applying our inclusion criteria, our analysis included 63,525 middle-aged and older-aged adults from all states and UTs in India.

### 2.5 Statistical methods

We determined the crude and weighted prevalence of SFI (and 95% Confidence Intervals) in all 36 states and UTs in India. We also utilized Multivariable Logistic Regression Analysis to determine salient correlates. In addition, we considered the interaction effects between socioeconomic status – education and household monthly per capita expenditure as two proxies – and geographic factors: urban-rural residence and geographic region. We tested for the overall significance of each interaction effect using an adjusted Wald Test. We used sampling weights for all analyses to account for LASI's complex survey design effects. Stata 18.5 was used for data analysis.

## 3 Results

### 3.1 Sample characteristics

Of all 63,525 respondents, 54.1% were women, 64.9% were aged 45–64, and most were married (Table 2). Hindus and Other Backward Classes formed the sample's dominant religious and caste groups. Respondents lived in the country's six major geographic regions, ranging from 3.1% in the Northeast to 24.4% in the South. Additionally, 69.1% were inhabitants of rural areas. 57.4% were migrants who had moved at some point during their lifetime. Regarding the sample's socioeconomic status, 68.1% had completed primary school or less. The sample was evenly distributed across quintiles of household monthly per capita expenditure (hereafter referred to as household expenditure). While 46.4% of adults were working, 26.0% had never worked for more than three months in their lifetime.

**Table 2:** Sample distribution (*n*) and percentage distribution (%) of SFI across demographic and socioeconomic characteristics.

	Sample Distribution		Food Insecurity	
	*n*	%	%	95% CI
**Age Group**				
45–54 years	23,323	35.0	5.9	[4.9, 7.2]
55–64 years	19,553	29.9	6.3	[5.6, 7.2]
65–74 years	14,088	23.9	6.3	[5.6, 7.2]
75+ years	6561	11.2	6.1	[5.0, 7.3]
**Gender**				
Men	29,600	45.9	6.0	[5.4. 6.8]
Women	33,925	54.1	6.3	[5.4, 7.2]
**Marital Status**				
Married	47,305	73.4	5.6	[5.0, 6.4]
Widowed	14,059	23.7	7.6	[6.7, 8.6]
Other	2161	2.9	7.8	[5.2, 11.5]
**Religion**				
Hindu	47,255	83.3	5.8	[5.3, 6.3]
Muslim	6699	10.1	8.6	[5.6, 12.8]
Christian	6268	3.0	8.5	[5.1, 13.8]
Other	3303	3.6	5.6	[3.8, 8.0]
**Caste**				
Other Caste	16,100	24.8	4.1	[3.5, 4.9]
Scheduled Caste	10,910	19.8	9.3	[8.1, 10.7]
Scheduled Tribe	11,592	8.8	6.9	[5.7, 8.3]
Other Backward Classes	24,923	46.6	5.8	[4.8, 6.9]
**Geographic Region**				
North	11,593	12.5	2.7	[2.3, 3.2]
Northeast	8780	3.1	2.6	[2.0, 3.3]
East	11,012	22.6	7.0	[6.2, 8.0]
Central	8838	21.2	7.8	[6.5, 9.3]
West	8174	16.3	5.5	[4.6, 6.5]
South	15,128	24.4	6.6	[4.9, 8.9]
**Place of Residence**				
Rural	41,636	69.1	7.1	[6.5, 7.7]
Urban	21,889	31.0	4.2	[3.0, 5.8]
**Migration Status**				
Lived for whole life	27,652	42.7	6.5	[5.5, 7.7]
Lived for 10 years or more	33,254	53.8	5.9	[5.3, 6.5]
Lived for less than 10 years	2619	3.5	6.5	[4.9, 8.6]
**Highest Level of Education**				
No schooling	30,024	50.8	7.8	[7.1, 8.5]
1–5 years of schooling	11,645	17.3	6.5	[5.6, 7.6]
6–9 years of schooling	10,008	14.0	3.9	[3.3, 4.7]
10 years of schooling or more	11,848	17.9	3.0	[1.4, 6.2]
**Household Monthly per Capita Expenditure**				
Poorest	12,686	21.0	8.0	[7.2, 9.0]
Poor	12,794	21.2	6.7	[5.9, 7.7]
Middle	12,763	20.3	5.7	[4.9, 6.7]
Richer	12,816	19.5	4.8	[4.0, 5.7]
Richest	12,466	18.0	5.3	[3.5, 7.9]
**Working Status**				
Currently working	29,233	46.4	6.8	[5.9, 7.9]
Currently not working	16,767	27.7	7.1	[6.0, 8.3]
Never worked	17,525	26.0	4.0	[3.5, 4.7]

The prevalence of SFI ranged from five percent to eight percent across age groups, gender, marital status, and migration status, and there was no significant variation within subgroups of each of these factors. However, Muslim and Christian respondents had a higher prevalence compared to their Hindu counterparts, and there was a 5.1 percentage point difference in SFI between members of the Scheduled Castes and the Upper Castes. Addressing geography, the Central, East, and South regions had the highest proportion of severely food-insecure adults, namely 7.8%, 7.0%, and 6.6%, respectively. We also found a nearly three percentage point difference in the proportion of severely food-insecure adults among rural and urban dwellers.

SFI was also patterned along socioeconomic lines. As the level of educational attainment increased, there was a decline in food insecurity. Those with ten years of schooling or higher had a 4.8 percentage point decrease relative to those who had not attended school in their lifetime. A similar trend was apparent for household expenditure. However, the difference between subcategories was not as high as education: there was a 2.8 percentage point difference in SFI comparing the “poorest” household expenditure quintile to the “richest.”

### 3.2 Geographic distribution

For adults aged 45 and above in India, we estimated that 6.2% reported SFI (95% CI: 5.6, 6.8). We also found notable weighted state and UT-based differences in the extent of SFI (Table 3). Across states/UTs, the prevalence was the highest in Madhya Pradesh (10.3%), followed by Jharkhand (8.2%), Bihar, and Tamil Nadu (both 7.8%). Additionally, the prevalence was higher than the national estimate for eight states, which were regionally patterned by geographic region: Central (Madhya Pradesh, Uttar Pradesh), East (Bihar, West Bengal, and Jharkhand), and South (Tamil Nadu, Karnataka, and Andhra Pradesh). Among 23 states/UTs, the weighted SFI was less than or equal to 5.0%. The lowest-ranking states and UTs in the country were Nagaland (0.2%), Goa (1.5%), Arunachal Pradesh, and Lakshadweep (1.6%).

**Table 3:** The weighted and unweighted prevalence of severe food insecurity (%) and 95% confidence intervals (CI) for India and 36 States/Union Territories.

State/UT	Region	Unweighted	95% CI	Weighted	95% CI
India		4.8	[4.7, 5.0]	6.2	[5.6, 6.8]
**States**					
Andhra Pradesh	South	6.1	[5.2, 7.1]	6.6	[4.7, 9.1]
Arunachal Pradesh	Northeast	1.4	[0.8, 2.5]	1.6	[0.7, 3.4]
Assam	Northeast	2.3	[1.7, 3.2]	2.4	[1.6, 3.4]
Bihar	East	8.0	[7.2, 9.0]	7.8	[6.2, 9.8]
Chhattisgarh	Central	2.0	[1.4, 2.7]	2.0	[1.4, 2.9]
Goa	South	1.3	[0.8, 2.2]	1.5	[0.8, 3.0]
Gujarat	West	6.2	[5.3, 7.4]	5.4	[4.4, 6.7]
Haryana	North	3.0	[2.3, 4.0]	3.0	[2.1, 4.3]
Himachal Pradesh	North	1.7	[1.1, 2.6]	2.2	[1.2, 4.1]
Jammu and Kashmir	North	2.0	[1.4, 2.9]	1.8	[1.0, 3.2]
Jharkhand	East	7.5	[6.5, 8.7]	8.2	[5.9, 11.2]
Karnataka	South	5.9	[4.9, 7.0]	7.0	[3.2, 14.6]
Kerala	South	3.7	[2.9, 4.5]	3.4	[2.1, 5.5]
Madhya Pradesh	Central	9.2	[8.1, 10.3]	10.3	[7.5, 13.8]
Maharashtra	West	5.2	[4.5, 6.0]	5.5	[4.3, 7.0]
Manipur	Northeast	2.5	[1.8, 3.5]	2.1	[1.2, 3.5]
Meghalaya	Northeast	2.3	[1.5, 3.6]	2.7	[1.3, 5.7]
Mizoram	Northeast	4.6	[3.5, 6.0]	4.3	[2.1, 8.3]
Nagaland	Northeast	0.4	[0.2, 1.0]	0.2	[0.1, 0.7]
Odisha	East	4.8	[4.0, 5.7]	4.5	[3.5, 5.8]
Punjab	North	3.9	[3.1, 4.8]	4.0	[2.8, 5.5]
Rajasthan	North	1.8	[1.3, 2.5]	1.8	[1.2, 2.6]
Sikkim	East	2.5	[1.7, 3.7]	2.8	[1.4, 5.5]
Tamil Nadu	South	6.5	[5.7, 7.4]	7.8	[6.0, 10.0]
Telangana	South	4.8	[4.0, 5.7]	4.4	[3.2, 6.0]
Tripura	Northeast	5.0	[3.9, 6.6]	5.1	[3.0, 8.4]
Uttar Pradesh	Central	7.4	[6.6, 8.2]	7.4	[6.2, 8.8]
Uttarakhand	North	4.5	[3.5, 5.8]	4.1	[2.8, 5.9]
West Bengal	East	6.2	[5.4, 7.2]	7.1	[5.7, 8.9]
**Union Territories (UTs)**					
Andaman and Nicobar	South	2.6	[1.7, 3.9]	2.2	[1.1, 4.1]
Chandigarh	North	2.2	[1.4, 3.4]	2.1	[1.2, 3.6]
Dadra and Nagar Haveli	West	5.5	[4.2, 7.1]	5.3	[3.8, 7.4]
Daman and Diu	West	3.2	[2.2, 4.7]	3.6	[2.3, 5.6]
Lakshadweep	South	1.5	[0.9, 2.5]	1.6	[0.8, 3.2]
NCT Delhi	North	4.8	[3.7, 6.2]	4.1	[2.7, 6.1]
Puducherry	South	5.2	[4.1, 6.5]	5.6	[3.9, 8.0]

### 3.3 Demographic and socioeconomic correlates

After adjusting for pertinent covariates in a Multivariate Logistic Regression Analysis, we found that age group, gender, and migration status were not significantly associated with SFI (Table 4). However, marital status, religion, caste, geographic region, and urban-rural residence were noteworthy correlates. Being widowed, a member of a Scheduled Caste, and being Muslim were all associated with a higher risk of SFI. Notably, members of Scheduled Castes were 1.78 times more likely to be food insecure than upper-caste individuals (95% CI: 1.32, 2.39). Additionally, the likelihood of food insecurity among Muslims was 1.82 times that of Hindus (95% CI: 1.06, 3.13).

**Table 4:** Multivariate Logistic Regression Analysis of the associations between demographic and socioeconomic covariates and severe food insecurity.

	Odds Ratio [95% CI]
**Age Group**	
45–54 years	**ref**
55–64 years	1.00 [0.84, 1.18]
65–74 years	0.92 [0.77, 1.10]
75+ years	0.81 [0.61, 1.08]
**Gender**	
Men	**ref**
Women	1.08 [0.60, 1.93]
**Marital Status**	
Married	**ref**
Widowed	1.32 [1.08, 1.60] ∗∗
Other	1.43 [0.92, 2.22]
**Religion**	
Hindu	**ref**
Muslim	1.82 [1.06, 3.13] ∗
Christian	1.50 [0.83, 2.70]
Other	1.30 [0.89, 1.90]
**Caste**	
Other Caste	**ref**
Scheduled Caste	1.78 [1.32, 2.39] ∗∗∗
Scheduled Tribe	1.20 [0.82, 1.75]
Other Backward Classes	1.09 [0.84, 1.40]
**Geographic Region**	
North	**ref**
Northeast	0.92 [0.64, 1.30]
East	2.50 [1.98, 3.14] ∗∗∗
Central	2.84 [2.14, 3.77] ∗∗∗
West	2.14 [1.62, 2.83] ∗∗∗
South	2.63 [1.98, 3.48] ∗∗∗
**Place of Residence**	
Rural	**ref**
Urban	0.73 [0.57, 0.94] ∗
**Migration Status**	
Lived for whole life	**ref**
Lived for 10 years or more	0.89 [0.62, 1.29]
Lived for less than 10 years	1.18 [0.60, 2.33]
**Highest Level of Education**	
No schooling	**ref**
1–5 years of schooling	0.93 [0.75, 1.16]
6–9 years of schooling	0.61 [0.45, 0.81] ∗∗
10 years of schooling or more	0.50 [0.21, 1.17]
**Household Monthly per Capita Expenditure**	
Poorest	**ref**
Poor	0.86 [0.73, 1.02]
Middle	0.79 [0.64, 0.97] ∗
Richer	0.69 [0.55, 0.87] ∗∗
Richest	0.87 [0.62, 1.22]
**Working Status**	**ref**
Currently working	1.03 [0.78, 1.36]
Currently not working	0.59 [0.41, 0.85] ∗∗
Never worked	
**Constant**	0.03 [0.02, 0.05] ∗∗∗

Exponentiated coefficients rounded to one decimal point: 95% confidence intervals in brackets. The asterisks refer to the following p-values: ∗*p* < 0.05, ∗∗*p* < 0.01, ∗∗∗*p* < 0.001.

Findings from the Logistic Regression also support the state-based differences in SFI presented in Table 2. Notably, residents of the Central, East, and South geographic regions had a likelihood of SFI equal to or greater than twice the likelihood among residents in the North Geographic region. Moreover, urban-dwelling adults were 27% less likely to be severely food insecure than their rural counterparts (OR = 0.73; 95 CI: 0.57, 0.94). Additionally, SFI was patterned by educational attainment, household expenditure, and working status. Those with 6–9 years of schooling had a lower likelihood of SFI than respondents without schooling. For those who received 6–9 years of education, the likelihood of SFI was 39.3% lower than that of respondents who received no schooling (OR = 0.61; 95% CI: 0.45, 0.81). As the level of household expenditure increased, the likelihood of SFI significantly decreased, except for the “richest” wealth quintile. Those who had never worked had a lower likelihood of SFI, which was 0.59 times the likelihood of SFI among current workers (95% CI: 0.41, 0.85).

### 3.4 Interaction effects

**Fig. 2:**
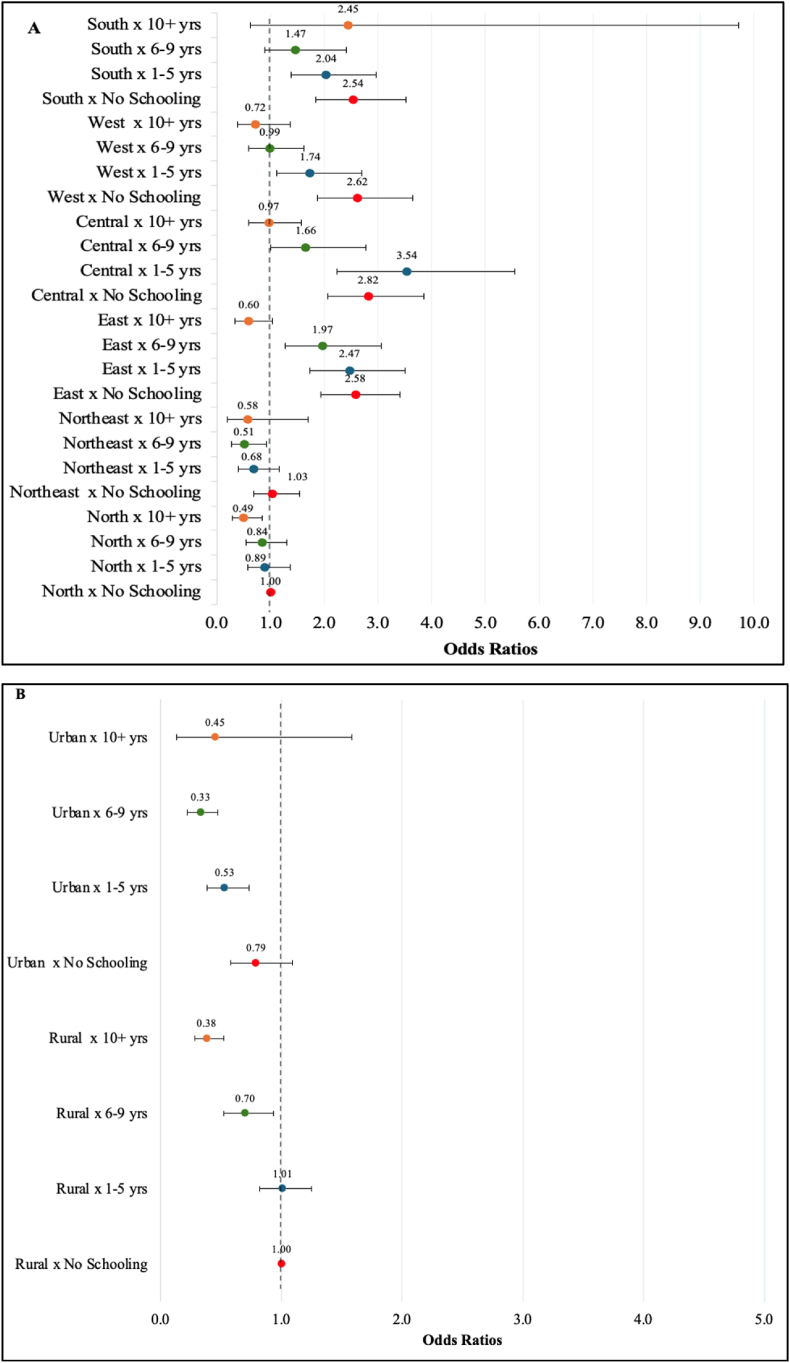
The estimated associations (odds ratios) between educational attainment and severe food insecurity stratified by geographic region (A) and place of residence (B).

Lastly, we tested whether geography (urban-rural residence, geographic region) modifies the relationship between socioeconomic status (educational attainment and household expenditure quintile) and SFI. We found significant interaction effects between education and urban-rural residence (p = 0.031) and education and geographic region (p = 0.032), but neither for household expenditure quintile. We depict our findings of the estimated likelihood of SFI across subgroups, with those without schooling in North India as our reference group (Fig. 2).

Across geographic regions, there was a graded decrease in the likelihood of SFI as the level of education increased (Fig. 2A). However, compared to the reference group, adults with no schooling or 1–5 years of education in the Central, East, and South regions had a greater likelihood of SFI. Comparing trends in the three regions, however, a higher likelihood of SFI persisted even for those with ten years of schooling or higher in the East region but not the other two. Additionally, the likelihood of SFI decreased as the level of education increased in both rural and urban India (Fig. 2B). However, this decrease in likelihood was more substantial in urban areas for adults with 1–5 years and 6–9 years of schooling (compared to the reference group).

## 4 Discussion

### 4.1 National and state prevalence

We estimated that the national prevalence of SFI among Indian adults aged 45 and above is 6.2%. According to nationally representative surveys, the prevalence of SFI among adults aged 50 and above in South Africa, Ghana, and Canada has been estimated to be 20.8%, 8.8%, and 4.5%, respectively (Gyasi et al., 2020; Koyanagi et al., 2019; Men and Tarasuk, 2020). Among households headed by adults 60 years or older, the prevalence of severely food-insecure households in Brazil is estimated to be 3.5% (Ribeiro et al., 2023). The prevalence we propose lies in the middle of all these countries. However, these national studies on adult SFI use different definitions and scales and differ in the study population, inclusion criteria, and time of data collection, making cross-country comparisons challenging. Apart from the issue of comparability, misreporting among adults of specific social groups in India is very likely, which we discuss in Section 4.3.

Additionally, we find a varied prevalence of SFI across India's states and UTs. Various factors likely contribute to this variation, including state capacity, population density, and implementation of food security programs. The North Indian states of Haryana and Punjab are food surplus states (Swaminathan and Bhavani, 2013), which may contribute to low SFI in the Northern region, as our results indicate. As per our estimations, Madhya Pradesh, Uttar Pradesh, West Bengal, Bihar, and Jharkhand had a high prevalence of SFI. These states in Central and Eastern India have the highest rates of multidimensional poverty, which shapes their high rank in undernutrition and stunting among children in addition to adult SFI (Alkire et al., 2021; Kandapan et al., 2023).

However, issues surrounding access are also essential to acknowledge. Notably, India's most extensive subsidized food provision program covering 800 million Indians, called the Public Distribution System (PDS), is operationalized at the state level (George and McKay, 2019, Rahman et al., 2018). While some states, such as Tamil Nadu and Chhattisgarh, have achieved impressively high coverage rates and minimal leakages, others have fallen behind (Dreze et al., 2019). As per 2020 population predictions, the PDS under-coverage of state populations is as high as 30.6% in Uttar Pradesh and 17.5% in Bihar (Khera and Somanchi, 2020). States and UTs that we estimated to have lower SFI, such as Goa, Nagaland, and Lakshadweep Islands, have the lowest predicted under-coverage rates. Additionally, leakages of PDS grains and the irregular provision of food rations have been documented in Bihar and Jharkhand (Dreze et al., 2019).

Barring this general perspective, many questions about the subnational variation of adult SFI still need to be answered. For instance, what are the drivers of SFI in southern India? Indeed, it is noteworthy that more well-off states, such as Tamil Nadu and Karnataka, were highly ranked in SFI. This is especially perplexing because Tamil Nadu has universal PDS coverage (Dreze et al., 2019).

### 4.2 A sociodemographic perspective

In addition to state/UT-level variation, we found differences within socioeconomic and demographic groups. Our results suggest that SFI is associated with socioeconomic disadvantage; we found a graded decrease in SFI as education increased. Education is a salient predictor of SFI in India (McKay et al., 2023). This is due to its close links with employment opportunities, wages, and the means to procure adequate food and nutrition. Expectedly, in line with education, the general trend indicated that individuals in lower wealth quintiles were more likely to be severely food insecure. With rising food prices and shocks such as the COVID-19 pandemic, the vulnerability of low-income communities is a significant concern.

We also found that widowed individuals were more likely to report SFI than married individuals. LASI findings indicate that widowed men and women are more likely to report food insecurity compared to their married counterparts due to economic vulnerabilities (Hossain, 2024). Relatedly, having a husband who is the primary breadwinner may be a protective factor against SFI for married women who do not work. This may be supported by our finding that those who had not worked for more than three months in their lifetime-of whom 61% were married women-were less likely to be food insecure than working individuals.

In India, one out of two Scheduled Caste individuals and one out of three Scheduled Tribe and Muslim individuals are estimated to be multidimensionally poor (Alkire et al., 2021). Our results indicate that Muslims and members of Scheduled Castes were especially vulnerable to being severely food insecure. Relatedly, food expenditures for Scheduled Caste and Muslim households are higher than those for Upper Caste and Hindu households respectively (Kaicker et al., 2022), and income disparities likely partially explain food security disparities. For both these social groups, inadequate caloric consumption has been documented (Bhuyan et al., 2020; Mahadevan and Suardi, 2013). In addition to structural inequities in income and education, members belonging to Scheduled Castes are disproportionately excluded from the PDS and other government schemes (Mahadevan and Suardi, 2013; Thorat and Lee, 2005). This can manifest as the exclusion of Scheduled Caste-majority neighborhoods in food ration eligibility or discrimination in the quantity or price of food rations (Thorat and Lee, 2005).

Additionally, we identified that adults in rural India were more likely to be severely food insecure than their urban counterparts. Prior studies have documented a similar difference for other markers of food insecurity and malnutrition among children and older adults (Fong, 2023; Kandapan et al., 2023; Subramanian et al., 2023). Strengthening government initiatives such as the Mahatma Gandhi National Rural Employment Guarantee Act and the boosting PDS coverage have likely reduced poverty and food insecurity in rural areas (Datt et al., 2019; Dreze et al., 2019). However, rural areas face unique challenges concerning food insecurity. Rural inhabitants are more likely to depend upon agriculture for their subsistence and have been disproportionately affected by climate change, compromised agricultural productivity, government food pricing, and inefficient grain storage (Dreze et al., 2019; Jaacks et al., 2021; Raj et al., 2022). Furthermore, a sizeable proportion of rural farmers and agricultural laborers tend to be landless and are, therefore, net consumers of food and likely face food shocks during volatile market prices and food shortages (Datt and Ravallion, 1998).

Additionally, we found that respondents with higher education levels in urban areas were more protected against SFI than their rural counterparts. The combination of contextual factors mentioned earlier may likely shape this trend. Addressing the compounded effects of geographic region and education level, inhabitants of the Central, East, and South regions with lower levels of education had a higher likelihood of SFI relative to less-educated inhabitants of the North. Various structural factors discussed in Section 4.1 may play a more significant role at the state or regional level in shaping SFI than individual factors indicative of privilege and wealth, such as educational attainment and income, which must be further investigated.

Notably, we identify a synergistic effect of education and geography but not household monthly per capita expenditure. Household expenditure as a proxy for socioeconomic status could fall short in capturing food expenditure patterns across income groups, the full extent of household wealth (including income and savings), and the intrahousehold allocation of resources, including money and food (Beshears et al., 2018; Harris-Fry et al., 2017; Swathysree et al., 2023).

### 4.3 Limitations

Our study has several limitations. First, we do not provide a holistic picture of food insecurity among adults in India; the trends related to mild and moderate food insecurity may differ from what we discuss. Second, food security was self-reported, which may have led to underreporting or overreporting. One possible reason driving this is social desirability bias. Prior global evidence has indicated that respondents may underreport or overreport food consumption to preserve individual and family honor, to avoid shame, or possibly in the hope of being included in a welfare program (Tadesse et al., 2020; Wheeler et al., 2019). In the Indian context, for instance, it has been found that malnutrition deaths are often underreported, while immunization is overreported (Saxena, 2018). It could be that women, older adults, migrants, and even individuals belonging to low-income and low-caste communities might have underreported food insecurity, but this claim requires further investigation.

Additionally, there could have been a differential consensus surrounding how much food is “enough food” for consumption and whether the quantity or size of meals was reduced due to food shortages or other factors among the sample's respondents, such as individual preferences or cultural norms. For instance, women in India likely eat less food as a cultural expectation and may change their food consumption based on household food availability without intentionally being aware of doing so (Harris-Fry et al., 2017). Additional research must consider measurement tools more suited to the Indian context which can account for self-reporting biases.

Furthermore, we could only capture cross-sectional insights; the LASI data for most states were collected during 2017-18. A dramatic change that has exacerbated SFI in India since 2017-18 is the COVID-19 pandemic. A study of 5000 respondents across India from April to May 2020 revealed that 66% had lost their employment, and 80% of households reduced their food intake (Kesar et al., 2021). A 200-district survey of farmers revealed that nearly one in four had yet to harvest crops due to lockdown-related reasons (Jaacks et al., 2021). Significantly, food insecurity during the COVID-19 pandemic affected different social groups disproportionately with rural-urban differences: households with a female or older adult head, individuals who received no schooling, members of Scheduled Castes, and Muslims were more likely to incur higher food expenditures (Kaicker et al., 2022). Even 22 months after the start of the COVID-19 pandemic, poverty rates had still not returned to pre-pandemic levels (Jha and Lahoti, 2022), which has repercussions for SFI today. The disproportionate burden in SFI related to religious identity, caste, education, monthly expenditure, and geographic region that we highlight has likely only magnified. At the same time, we expect new trends: women faring worse than men and possible differences in SFI rates between migrants and non-migrants.

### 4.4 Conclusion and broader implications

Through this study, we provide important context on the geographic and social distribution of SFI in India. We highlight specific states and regions with high SFI and identify at-risk groups. Our findings indicate that a decentralized or regional approach to alleviating SFI at the state/UT level is critical. This can be achieved by combating food access and affordability disparities and focusing on vulnerable socioeconomic groups in rural areas and the country's Central, Eastern, and Southern states. Strengthening government schemes for employment generation and agricultural support may reduce SFI in rural India. The introduction of the Pradhan Mantri Kisan Samman Nidhi (PM-KISAN) initiative is one avenue that has diminished the financial burden of small and marginal farmers with the larger aim of boosting agricultural output (Varshney et al., 2020). Additionally, existing targeted interventions and programs such as the PDS must address food security disparities along religious and caste lines. Owing to the subsequent impacts on health and quality of life associated with SFI, access to affordable food must be universally guaranteed for all Indians, particularly for middle-aged and older adults belonging to at-risk communities.

While we cannot establish the underlying causes of SFI, our findings establish many crucial links between sociodemographic factors and adult SFI and open new avenues for further investigation. Prospective population-level and qualitative research can examine middle-aged and older adults' unique challenges in affording, accessing, and storing food at state/UT and substate levels. Additionally, further research can identify approaches, interventions, and programs that have helped reduce SFI within the past decade, especially during the COVID-19 pandemic. Additional research must examine regional trends in SFI in specific geographic regions, namely Central, East, and South. The nexus between gender, poverty, marital status, working status, and adult SFI in India is another prospective area of exploration.

Another valuable area of inquiry is examining the relative contribution of contextual versus compositional factors in explaining the geographic variation in SFI at the individual, household, community, and state levels. We could not look at several compositional factors, some of which likely play a crucial role in SFI, including occupation, household size, monthly income, and health status. On the other hand, various contextual factors, including PDS coverage, the robustness of food ration operations, the effects of climate change, transportation infrastructure, and neighborhood of residence, also likely play a crucial role, which requires further investigation. Such a multilevel perspective can help identify intervention points at individual, community, and state levels.

## CRediT authorship contribution statement

**Ravi Sadhu:** Writing – original draft, Investigation, Formal analysis, Data curation. **Rockli Kim:** Writing – review & editing, Investigation, Conceptualization. **S.V. Subramanian:** Writing – review & editing, Supervision, Investigation, Conceptualization.

## Declaration of competing interest

The authors declare that they have no known competing financial interests or personal relationships that may have influenced the work reported in this paper.

## Data Availability

The IIPS LASI Wave 1 dataset is available at https://www.iipsindia.ac.in/content/LASI-data.
